# Wireless multihop backhauls for rural areas: A preliminary study

**DOI:** 10.1371/journal.pone.0175358

**Published:** 2017-04-12

**Authors:** Zainab Zaidi, Kun-chan Lan

**Affiliations:** 1 Independent Researcher, London, United Kingdom; 2 Department of Computer Science and Information Engineering, National Cheng Kung University, Tainan City, Taiwan; University of Texas at San Antonio, UNITED STATES

## Abstract

Rural areas have very low revenue potential. The major issue in providing low-cost broadband to rural areas is to provide reliable backhaul connections that spread over tens or even hundreds of miles, connecting villages to the nearest service provider. Along with aerial networks of Google and Facebook, there has been a considerable amount of research toward long-distance terrestrial WiFi links. As a comparison, WiFi routers are easier to be deployed and maintained by non-technical people from the local communities, whereas the aerial networks require professional support to operate. Moreover, they are still in the experimentation phase. However, the long distance WiFi links require high-gain directional antennas and very expensive tall towers for high data rates. On the other hand, multihop paths with stronger links may provide better data rates without the need of tall towers. In this paper, we evaluated the concept of using such multihop WiFi links for long backhaul connections. Our simulation results show that these networks can possibly be a cost-effective and practical solution for rural connectivity. These initial results can serve as a first step to understand the comprehensive feasibility of using multihop WiFi networks for backhaul connections in rural area.

## Introduction

In the modern telecom era of low-cost and high-speed WiFi technology, plug-and-play type small cells, flexible and programmable software defined networks and functional virtualization, there exists huge opportunity for bridging digital divide which never existed before [1]. Although, the advancements are mostly driven by high-capacity demands in urban areas, but low-cost, flexible use, and hardware sharing possibilities of these equipment could be the much-awaited enablers for a practically realizable and economically feasible first generation of rural network technology.

The major inhibitor for rural deployment is the return potential of the sparsely populated low-income communities. The usual distance that needs to be covered for providing backhaul connection, i.e., connecting remote villages to the nearest service provider, could be tens or hundreds of miles. According to [2], the revenue potential for a traditional wireless carrier in a least populated area of USA drops to $262 per square mile from $248,000 per square mile in a major urban center. When translated to low-income developing regions, the potential drops even further. Satellite, although provide global coverage, is also the most expensive approach, with the cost of 1 Mbits/s connectivity in Africa exceeding $3000/month [3]. Recent initiatives from Google (www.google.com/loon) and Facebook (www.internet.org) have started to look into exploiting the aerial space over the remote areas to provide connectivity. In aerial networks, a costly node/network could be made affordable as it serves a much larger area but economical network maintenance and providing always-on, reliable, and secure connectivity in wake of intermittent link/node failures are still big open challenges. Moreover, with 60% global population still on the other side of digital divide, it is a huge problem with no simple solution yet in sight. It is very important to explore possibilities and cheaper alternatives, specially those which can be managed and run by local communities.

On the other hand, there has been a considerable amount of research directed towards long-distance WiFi links [3–6], and the practicality of this approach has been successfully demonstrated, with the current record being a 6 Mbits/s link over 384 Km in Venezuela. Such links require LoS (Line of Sight) for sufficient signal-strength; otherwise, signal attenuation in the 2.4-5 GHz range becomes too high beyond a few hundred meters [4]. To provide LoS, significant infrastructure investments in terms of antenna towers are required [4]. The tall towers are usually one or two orders of magnitude more expensive than the equivalent radio equipment, e.g., a 45 m tower costs around $5000 according to [4]. The use of high-gain directional antennas also considerably increases the cost. A detailed survey of different rural communication initiatives is given in Section Related Work.

**Fig 1:**
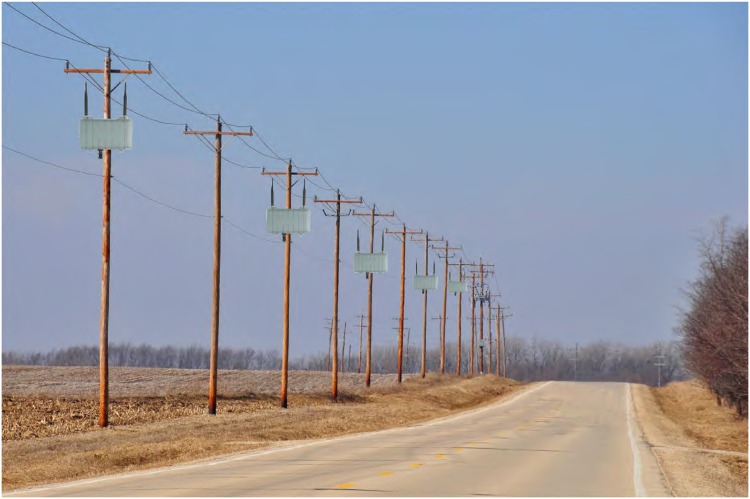
A road-side chain backhaul network.

In this paper, we focus towards multihop relay networks rather than a single link to provide real-time backhaul connectivity over large distances. Our main motivation comes from the fact that multihop paths with smaller and stronger links with high capacity may provide better data rates than a longer and weaker link with low-rate modulation. With low cost hardware such deployment may also be economical and cost-effective. With plug-and-play type of nodes, the local manpower can be trained to perform day-to-day maintenance tasks, which reduces the cost and delays in maintenance and also creates opportunities for involvement of local community, a must for sustained success of such project according to [7]. Such networks can be deployed along existing roads, as shown in Fig 1. They would be easily accessible and can utilize the available utility poles and resources, e.g., power supply and maintenance logistics, to further reduce the cost. These networks can be used to provide connectivity to the commuters as well along with relaying backhaul traffic, although, this scenario is outside the scope of present paper and our analysis considers only the backhaul traffic for reliable delivery. Commuter traffic will provide significant interference and reduce the capacity of network for relaying backhaul data.

As software defined networks with functional virtualization are becoming a reality, it is also possible that infrastructure could be deployed by a third party, e.g., government or charity organizations, and multiple service providers can launch their services and pay for the use of the infrastructure. In this paper, our analysis considered ISM band and 802.11 standards as a natural choice for cost-effective networks but similar results can be obtained for other licensed bands and standards.

The major concerns for such networks would be to provide economical and reliable connectivity in wake of excessive link/node failures in hostile environment along with quality of experience and security which are not analyzed in this study. A single node failure in linear topology can disconnect the network. Unavailability of reliable power supply and breakdowns further aggravate the vulnerability of network. Moreover, lack of locally available skilled human resources would delay the repairs and incur additional costs comparing with urban areas. A practical rural network should have sufficient self-healing mechanisms to cater for the excessive failures. We propose the use of redundant nodes and opportunistic routing to exploit spare wireless connections, i.e., if a node fails, two neighboring nodes can by-pass it and connect with a weaker link. The idea of using some nodes and links as backups for fault tolerance is also discussed in [4]. They used RF switches such that the backup node can only be used when needed and the normal operation is not affected when there is no fault. However, in the case of climate disasters, power breakdowns, or failures affecting a region, the redundant nodes may also fail.

We performed a preliminary analysis using theoretical models to evaluate the feasibility of such networks in terms of connectivity and cost. The actual deployment of such an approach should also consider specific environmental/terrain factors and other design issues, such as security and the availability/non-availability of alternative power sources. Our analysis shows very promising results of reliable connectivity when node failures affect individual nodes and also multiple nodes in a region. The study also shows that there exists an optimal setting for the considered scenarios where further addition of nodes in the network does not enhance reliability, i.e. probability of successful packet delivery. This optimal setting is used to estimate the cost for multihop backhaul chain networks. In this paper, we use multihop chains, chain networks, and linear networks interchangeably to describe wireless nodes in a linear topology.

This paper extends our earlier work [8], where the probability of existence of active multihop path was calculated using signal propagation model but without considering specific routing protocol, data rate, and correlated node failures. In this paper, we have used a version of opportunistic routing to exploit the redundant wireless paths while calculating the probability of packet delivery. We also tried to compare the effects of routing overhead over the performance of both routing regimes, i.e., opportunistic and traditional routing. Moreover, we have used node failures correlated in time and space as well as independent failures in our study.

The major contributions of this paper are the following.

1. We have shown that WiFi multihop chain networks have great potential to be used as long backhaul connections for rural areas. The performance is measured in terms of reliability in wake of intermittent link/node failures using theoretical models for radio propagation.
2. It is shown that improved robustness of chain networks can be achieved by using opportunistic forwarding methods and by adding redundancies in network topology.
3. The paper discusses the impact of overestimation of link strength in a theoretical analysis by comparing the results from field measurements.

The remainder of this paper is organized as follows: our system model and assumptions; the specific steps of the opportunistic forwarding scheme; the reliability of a single link, which is the building block of a wireless multihop chain network; and node failure models are presented in Section Preliminaries. Section Packet Delivery Probability derives recursive expressions for calculating the packet delivery probability for the different chain topologies considered in this study. Section Numerical Analysis presents detailed analysis of the reliability and cost of different topologies of chain networks. Section Related Work summarizes rural communication initiatives around the group and other relevant available literature. Finally, Section Conclusion concludes the paper.

## Preliminaries

### System model and assumptions

In this paper, the feasibility of multihop relay chain networks for long distance backhaul is evaluated in terms of probability of successful packet delivery over the backhaul. We create a hypothetical scenario which is generic enough for comprehensive evaluation and is sufficiently simple for computational tractability. The best deployment scenario for the specific region and available resources should be considered in a practical system. Our assumptions are as follows:

1. We assume identical and equidistant 802.11 a/b/g/n nodes spanning 100 km in this study. The choice of specific parameters are given in Section Numerical Analysis. Homogeneous network assumption allow us to write expressions for probability of successful delivery in closed form and provide a benchmark scenario which is helpful in analyzing the effects of heterogeneity in nodes and inter-nodal distances when required.
2. Most of the analysis in this paper uses 802.11g technology. We have shown some results from 802.11n for appreciation of increase in data rates with MIMO enhanced WiFi in Section Cost and Section Cost vs. Pole Length. Even better data rates can be achieved with 802.11ac. The results are not reported in the paper as it will only be a straightforward extension to the analysis.
3. We also assume a scheduled MAC for the realization of opportunistic routing. MAC-related issues are beyond the scope of the current paper, although we require ACKs from multiple potential forwarders in opportunistic routing. How this will be implemented within 802.11 nodes is left for future work. As a first step to understand the feasibility of using opportunistic routing for backhaul WiFi connections, we focus only on the discussion of routing issues in an ideal setting with low external interference and where proper scheduling with guard times in modified MAC for rural communication [5] can take care of collisions. The external interference, however, is likely to grow even in the remote areas with time and it would be more suitable to use SINR (Signal to Interference and Noise Ratio) instead of SNR (Signal to Noise Ratio) used in this paper. The direct implication would be reduction in the packet delivery probability because of additional collisions and reduced link capacity. As a result, performance measures reported in the paper would also become worse.
4. For signal propagation, we use Okumura-Hata model for free-space. Channel is AWGN (Additive White Gaussian Noise) with no multipath fading. Both assumptions are relevant for a number of remote and rural areas. According to [9], the typical rms delay spread, a measure for multipath richness, for open rural areas is around 0.1*μ*s, whereas, the typical value for urban environment is 1 − 2.5*μ*s. In highly forested and hilly areas, however, multipath fading should be considered where the rms delay spread can be as high as 5*μ*s.
5. Omni-directional antennas are assumed now but the actual deployment can use combination of directional antennas.
6. All nodes are aware of the topology and an estimated transmission range upper bound which can be done in the initial bootstrapping phase.

### Opportunistic routing

Opportunistic routing exploits the broadcast nature of the wireless medium. When a packet is forwarded, it generally reaches more than one node in a typical wireless mesh networking environment [10–12]. In traditional routing, only the forwarder selected by the routing algorithm keeps the packet, and the remaining nodes discard it. In opportunistic routing, however, the forwarder is not decided beforehand; instead, the node closest to the destination among the ones that have received the packet will forward it. A low-overhead agreement mechanism is typically employed to decide which node will forward the packet [12]. It is important to avoid duplicate forwarding to minimize packet collisions.

Different versions of opportunistic routing are proposed in the literature based on specific applications. A brief survey of opportunistic routing algorithms is given in Section Related Work. In our analysis, we used a version of geometric opportunistic routing as described in [12] along with scheduled transmissions. However, as we are interested in evaluating the reliability of connections, we have assumed that packets are forwarded individually rather than in batches to simplify the analysis. Also, ACKs are required from all potential forwarders scheduled in the priority order for each packet transmission. Priority order is selected according to the closeness to the destination. Moreover, multiple simultaneous non-interfering transmissions are allowed to occur.

**Fig 2:**
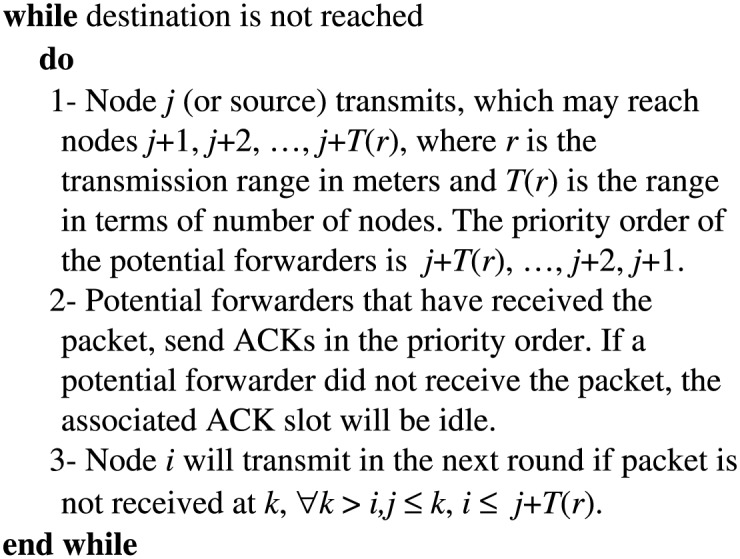
Opportunistic forwarding in wireless multihop chain network.

Our opportunistic forwarding procedure assumes that each node is aware of the topology of the wireless chain and has some estimate about the transmission range. This topology information can be provided in the initial bootstrapping phase of the network deployment and can be inferred from the node ID in the linear network. The nodes are assigned IDs from 0 to *n*, where 0 is the source and *n* is the destination. The range estimate can be initialized to a theoretically predicted value which can be updated after every successful forwarding step. This estimate can be supplied in the packet header to help the nodes determine their own positions in the priority list of potential forwarders. The steps of the opportunistic forwarding algorithm are given in Fig 2.

In this analysis, ACKs, in opportunistic routing as well as traditional routing, are assumed to have ideal reception. They are usually very small packets (14 bytes in 802.11) in comparison to data packets, which are usually over 1 Kbytes, and have much greater probability of successful delivery when compared with delivery probability of data packets. In practical scenarios, they could be dropped and collided with other ACKs which would adversely affect performance.

### Reliability of a wireless link

One of the fundamental elements of our analysis is the probability of successful delivery of a packet over a wireless link considering the effects of the radio propagation environment. We have followed a methodology similar to [13], although our study is mostly relevant to suburban or free-space scenarios and we are considering LoS (Line of Sight) only. In this paper, we used the empirical Okumura-Hata model for free space path loss [14]. Most of the empirical path loss models proposed for wireless communications [14] are useful for cellular settings, i.e., 100-2000 MHz range and antenna heights of 30 m. On the other hand, the models developed and adjusted for WLANs assume antennas placed at 1-2 m above the ground [15], which is also not true for our analysis. The probabilities of packet delivery using the Okumura-Hata model with parameters suitable for our operating scenario are similar to the ones from a specific outdoor path loss model for 2.4 GHz [15] [8]. The basic propagation model with shadowing noise is as follows [14]:

$$\begin{array}{ccc} & & S{\left(d\right)}_{\left(\text{dBm}\right)}=\\ & & \;\;\;{\text{EIRP}}_{\left(\text{dBm}\right)}+10\log_{10}{\left(G_{r}\right)}_{\left(\text{dB}\right)}-L{\left(d\right)}_{\left(\text{dB}\right)}+\epsilon_{\left(\text{dB}\right)}, \end{array}$$ (1)

where $S{\left(d\right)}_{\left(\text{dBm}\right)}$ is the received power in dBm at a distance *d* from the transmitter, ${\text{EIRP}}_{\left(\text{dBm}\right)}$ is the effective isotropically radiated power in dBm, *G*_*r*_ is the receiver antenna gain, $L{\left(d\right)}_{\left(\text{dB}\right)}$ is the path loss at distance *d* in dB, and $\epsilon_{\left(\text{dB}\right)}$ is the log-normal shadowing noise with standard deviation *σ*_*ϵ*_. In this analysis, ${\text{EIRP}}_{\left(\text{dBm}\right)}$ is taken as 36 dBm for 2.4 GHz, which is the maximum allowable point-to-point value from the FCC. It is reasonable to assume that the antennas used for transmission will also be used for the reception of signals and that the maximum allowable transmitter antenna gains from the FCC can be used as the values of $10\log_{10}{\left(G_{r}\right)}_{\left(\text{dB}\right)}$, i.e., 6 for 2.4 GHz. The typical value for *σ*_*ϵ*_ for free space is 8 dB [14]. According to the Okumura-Hata (OH) model, the path loss $L{\left(d\right)}_{\left(\text{dB}\right)}$ in Eq (1) for free space is as follows [14]:

$$\begin{array}{c} L{\left(d\right)}_{\left(\text{dB}\right)}=A_{{\;}_{OH}}+B_{{\;}_{OH}}\log_{10}\left(d\right)-D_{{\;}_{OH}}, \end{array}$$ (2)

where *d* is the distance of the receiver from the transmitter in km. The OH model parameter values, as given in [14], for free space are: *A*_*OH*_ = 69.55 + 26.16 log_10_(*f*_*c*_) − 13.82 log_10_(*h*) − *a*_*OH*_(*h*), *B*_*OH*_ = 44.9 − 6.55 log_10_(*h*), and *D*_*OH*_ = 40.94 + 4.78[log_10_(*f*_*c*_)]^2^ − 18.33 log_10_(*f*_*c*_). Also, as given in [14], *a*_*OH*_(*h*) = [1.1 log_10_(*f*_*c*_) − 0.7]*h* − [1.56 log_10_(*f*_*c*_) − 0.8], where *h* is the height of the wireless nodes from the ground in m and *f*_*c*_ is the carrier frequency in MHz. A simplified expression for *S*(*d*) can be written as:

$$\begin{array}{c} S{\left(d\right)}_{\left(\text{dBm}\right)}=\alpha -\beta \log_{10}\left(d\right)+\epsilon_{\left(\text{dB}\right)}, \end{array}$$ (3)

where $\alpha ={\text{EIRP}}_{\left(\text{dBm}\right)}+10\log_{10}{\left(G_{r}\right)}_{\left(\text{dB}\right)}-A_{{\;}_{OH}}+D_{{\;}_{OH}}$ and *β* = *B*_*OH*_.

Under the assumption of minimal collisions, outage is the dominant error event [13]. If *P*_*outage*_ = Pr(*C* < *R*), where *C* is the capacity of the link and *R* is the data rate, both in units of Mbits/s/Hz, the probability of successful packet delivery will be

$$\begin{array}{c} P=1-P_{outage}=1-\mathrm{Pr}\left(C<R\right) \end{array}$$ (4)

The outage probability can also be defined in terms of the transmission range of the nodes [16]. Both definitions are correct, and equivalence can be easily shown. The link between the two nodes is assumed to be an AWGN channel [13], whose capacity is given as

$$\begin{array}{c} C=\log_{2}(1+\frac{S(d)}{\sigma^{2}}), \end{array}$$ (5)

where *S*(*d*) is the received power in watts and *σ*^2^ is the noise variance, given as *N*_0_/2 × *BW* [16]. For 802.11b/g BW=22MHz. Noise spectral density is given as $N_{0}=k_{B}T_{K}\;\text{W}/\text{Hz}$, where *k*_*B*_ is the Boltzmann constant and *T*_*K*_ is the temperature in Kelvin. Typically $T_{K}=300\;\text{K}$ or *N*_0_ ≈ 10^−21^ to yield typical noise floor value of -100dBm for 802.11 receivers [17].

After substitution of *C* from Eq (5) into Eq (4) and simplification, the probability of successful packet delivery *P*, also denoted as *P*(*R*, *d*), is given as

$$\begin{array}{c} P\left(R,d\right)=1-\mathrm{Pr}(S\left(d\right)<\left(2^{R}-1\right)\sigma^{2}). \end{array}$$

Changing *S*(*d*) in dBm,

$$\begin{array}{c} P\left(R,d\right)=1-\mathrm{Pr}(S{\left(d\right)}_{\left(\text{dBm}\right)}<10\log_{10}(10^{3}\left(2^{R}-1\right)\sigma^{2})). \end{array}$$ (6)

After substitution of $S{\left(d\right)}_{\left(\text{dBm}\right)}$ from Eq (3) and simplification

$$\begin{array}{ccc} P(R,d) & = & 1-\mathrm{Pr}(\epsilon_{\left(\text{dB}\right)}<10\log_{10}(10^{3}\left(2^{R}-1\right)\sigma^{2})-\alpha +\beta \log_{10}\left(d\right))\\ & = & 1-Q(\left\{10\log_{10}(10^{3}\left(2^{R}-1\right)\sigma^{2})-\alpha +\beta \log_{10}\left(d\right)\right\}/\sigma_{\epsilon }) \end{array}$$ (7)

where $Q\left(x\right)=\frac{1}{\sqrt{2\pi }}\int_{x}^{\infty }e^{-\frac{t^{2}}{2}}dt$. Note that the probability *P*(*R*, *d*) is the theoretical upper bound for an AWGN channel and that in practical systems, e.g., 802.11a/b/g/n, link probabilities are smaller than the one predicted by the above expression.

For MIMO transceivers with *N*_*T*_ transmitter antennas and *N*_*R*_ receiver antennas, the capacity of an AWGN link, with equal power allocation on all parallel sub-channels, is given as [18]:

$$\begin{array}{c} C=\min\left\{N_{T},N_{R}\right\}\log_{2}(1+\frac{N_{R}}{\min\{N_{T},N_{R}\}}\frac{S(d)}{\sigma^{2}}). \end{array}$$ (8)

Incorporating Eq (8) in Eq (4) yields the reliability of a wireless link with MIMO transceivers as

$$\begin{array}{ccc} & & P(R,d)=1-\\ & & \;Q(\{10\log_{10}(10^{3}\left(2^{R/N_{m}}-1\right)\frac{\sigma^{2}N_{m}}{N_{R}})-\alpha +\beta \log_{10}\left(d\right)\}/\sigma_{\epsilon }) \end{array}$$ (9)

where *N*_*m*_ = min{*N*_*T*_, *N*_*R*_}.

### Mathematical representation of node failures

It is difficult to predict the typical equipment failure rate. The likelihood of mechanical part failure, in general, increases with usage. Rural broadband trials have shown that the component failure rate in such settings is considerably greater than that in urban environments [19]. In this paper, generic models are used to represent the condition of inoperative nodes, which could result from a number of causes, e.g., power failures, intentional or unintentional human interventions, environmental factors, accidental damage, software/hardware malfunctions, protocol artifacts, such as 802.11 MAC association/re-association delays, and so on, in addition to the failure of mechanical parts. We consider the following three types of node failures.

#### Independent

The first, simpler model assumes *iid* node failures with probability *f*. Each node can fail with probability *f* at any instance, independent of failures in the past and also at other nodes. These failures primarily represent the temporary outages caused by protocol artefacts and power fluctuations, among others.

#### Correlated in time

In the second model, we assume that node failures are correlated in time and follow a Markov model. This model represents all node faults which last for some time, e.g., component failures, theft, accidental damages, software/hardware malfunctions, etc. Here,

$$\begin{array}{c} \mathrm{Pr}\left(F_{k}\left(i\right)|F_{k-1}\left(i\right)\right)=f_{t};\;\mathrm{Pr}\left(F_{k}\left(i\right)|F_{k-1}^{c}\left(i\right)\right)=f,\forall i \end{array}$$

where *F*_*k*_(*i*) denotes the failure of node *i* at time *k* and $F_{k}^{c}\left(i\right)$ denotes the event that node *i* is not in failure at time *k*. In general, *f* < <*f*_*t*_. The discrete time instant *k* refers to the *k*^*th*^ step of opportunistic routing or the discrete time slot, and details of this are given in the next section. The Markov model is general enough to model a rich combination of failure situations. Because state residence times in a discrete-time Markov chain obey geometric distributions, we can write the mean interval of node failure as *f*_*t*_/(1 − *f*_*t*_) in terms of discrete time instants. Different values of *f*_*t*_ can be chosen to model longer or shorter failure intervals.

#### Correlated in time and space

It might be reasonable to assume independence of failure from one node to another when a failure is caused by malfunctions or human interventions, but failures due to power breakdowns or climate, i.e., storm, strong winds, etc., affect a considerably larger region rather than a single node and last for some times. To model these, we assume Markov dependency in space and in time ∀*i*, i.e.,

$$\begin{array}{ccc} \mathrm{Pr}(F_{k}\left(i\right)|F_{k-1}\left(i\right),F_{k}\left(i-1\right)) & = & f_{ts};\\ \mathrm{Pr}(F_{k}\left(i\right)|F_{k-1}^{c}\left(i\right),F_{k}\left(i-1\right)) & = & f_{s};\\ \mathrm{Pr}(F_{k}\left(i\right)|F_{k-1}\left(i\right),F_{k}^{c}\left(i-1\right)) & = & f_{t};\\ \mathrm{Pr}(F_{k}\left(i\right)|F_{k-1}^{c}\left(i\right),F_{k}^{c}\left(i-1\right)) & = & f. \end{array}$$

Mostly, *f* < <*f*_*s*_, *f*_*t*_ < *f*_*ts*_. The values of *f*_*s*_ and *f*_*ts*_ can be chosen to model failures affecting a larger or smaller region.

A list of variables in our analysis and their respective definitions is given in Table 1 for quick reference. Some variables will be defined in the next section.

**Table 1:** Variables and their definitions.

Variable	Definition
*P*(*R*, *d*)	Probability of successful packet delivery over distance *d* with rate *R*
*σ*_*ϵ*_	Standard deviation of shadowing noise
*σ*	AWGN noise variance
*N*_*T*_/*N*_*R*_	Number or transmitter/receiver antennas
*f*	Probability of *iid* node failures
*f*_*t*_	Probability of nodes failures correlated in time
*f*_*s*_	Probability of nodes failures correlated in space
*f*_*ts*_	Probability of nodes failures correlated in time and space
*n*	Number of primary nodes in a chain network
*n*_*r*_	Number of total nodes in a hybrid chain
*m*	Extra node is provided to every *m*^*th*^ node in a hybrid chain
*X*_*k*_	Vector of size *n* or *n*_*r*_, *X*_*k*_(*i*) is the probability of node *i* to be a preferred forwarder in *k*^*th*^ step
*A*	*n* × *n* or *n*_*r*_ × *n*_*r*_ matrix, *a*_*ij*_ is the probability of reception from node *j* to node *i*
*P*_*D*_	Size *n* or *n*_*r*_ vector, *P*_*D*_(*i*) is the probability of reaching destination from node *i*
*T*_*R*_	Number of allowed transmissions
*D*	*n* × *n* or *n*_*r*_ × *n*_*r*_ diagonal matrix containing primary diagonal of *A*
*P*_*sc*_	Probability of successful packet delivery over a simple chain
*P*_*hc*_	Probability of successful packet delivery over a hybrid chain

## Packet delivery probability

**Fig 3:**
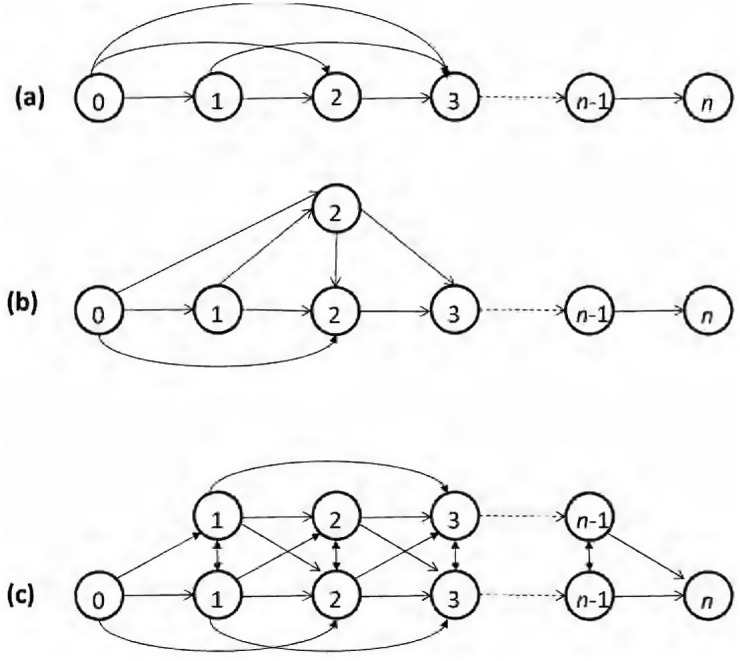
Different chain topologies. (a) simple chain, (b) hybrid chain, and (c) double chain.

Different topologies for multihop chain networks, as used in this study, are shown in Fig 3. A simple chain is the most common configuration of placing one node after another. We also introduced chain topologies with redundant nodes, called hybrid chains. In a hybrid chain, a redundant peer is provided to every *m*^*th*^ node, as shown in Fig 3. When *m* = 0, a hybrid chain becomes a simple chain, and when *m* = 1, we have redundant peers for every node, resulting in a trivial setting of placing two chains together instead of one, called a double chain, which is also used to benchmark the improvement in reliability. The redundancy should help in improving the reliability compared to a simple chain. Not all possible links are shown in Fig 3.

To simplify the feasibility analysis, we assumed that all nodes are identical, equidistant and erected at the same height. Networks deployed along a rural road with nodes mounted on utility poles should also have similar characteristics. However, the actual deployment may include heterogeneous nodes over different mounts suitable to the terrain and environment. In this paper, we are concerned only with the feasibility and practicality of long WiFi chain networks using generic operational settings.

### Simple chain

To calculate the probability of successful packet delivery *P*_*sc*_ over *n* nodes of a simple chain when the aforementioned opportunistic forwarding scheme is used, we define the following matrices. The source is referred to as node 0, and the destination is node *n*.

1. *X*_*k*_ is a vector of size *n*, where *X*_*k*_(*i*) is the probability of node *i*, 0 ≤ *i* ≤ *n* − 1 being a preferred forwarder in the *k*^*th*^ step. A node is a preferred forwarder if it has received the packet but nodes relatively closer to the destination do not. Initially, only the source has the packet to transmit, i.e.,
  $$\begin{array}{c} X_{0}\left(i\right)=\{\begin{array}{cc} 1, & i=0;\\ 0, & 1\leq i\leq n-1. \end{array} \end{array}$$
  For the subsequent steps *k*, *X*_*k*_ can be calculated using the packet reception probabilities and previous *X*_*k*−1_ as given later.
2. *A* is an *n* × *n* matrix, where *a*_*ij*_ is the probability of packet reception at node *i* when the transmitter is node *j* and nodes *l* > *i* do not receive the packet, i.e.,
  $$a_{ij}=P_{i-j}\prod_{l=i+1}^{j+M}\left(1-P_{l-j}\right),\;\;\;j\leq i\leq j+M,$$
  where *P*_*i*_ = *P*(*R*, *id*), *d* is the inter-nodal spacing, and *P*(*R*, *d*) is given in Eq (7) or Eq (9). *M* = min{*T*(*r*), *n* − *j*}. In our analysis, the transmission range *T*(*r*) is defined in terms of the probability of packet reception, i.e., *P*(*R*, *id*) ≥ 0.1, *j* ≤ *i* ≤ *j* + *T*(*r*).
3. *P*_*D*_ is a 1 × *n* vector, where *P*_*D*_(*i*) = *P*_*n*−*i*_, i.e., the probability of reaching the destination, node *n*, from node *i*.

At every step, *X*_*k*_ can be updated from *X*_*k*−1_ as follows

$$\begin{array}{c} X_{k}=\{\begin{array}{cc} AX_{k-1}, & 1\leq k<T_{R}\\ AX_{k-1}-D^{T_{R}}\left[A-D\right]X_{k-T_{R}-1}, & k\geq T_{R}. \end{array} \end{array}$$ (10)

where *T*_*R*_ is the number of allowed transmissions, including re-transmissions, and *D* is an *n* × *n* diagonal matrix containing the primary diagonal of *A*. The elements of vector [*A* − *D*]*X*_*k*−*T*_*R*_−1_ are the probabilities for each node being a preferred forwarder due to receiving a packet at the *k* − *T*_*R*_ − 1 step from any earlier node in the chain except itself. The probability of successful packet delivery *P*_*sc*_ over *n* nodes of a simple chain is

$$\begin{array}{c} P_{sc}=\sum_{k=1}^{nT_{R}}P_{D}X_{k}. \end{array}$$ (11)

It is interesting to note here that the Eqs (10) and (11) represent the evolution of *n*-state discrete time Markov chain with an absorbing state as the destination. Thus far, the calculations of *P*_*sc*_ have not considered node failures. If all nodes are assumed to have *iid* failures with probability *f*, we augment *P*_*i*_ in the formulation of matrices *A* and *P*_*D*_ given above as *P*_*i*_ = *P*(*R*, *id*)(1 − *f*) rather than *P*_*i*_ = *P*(*R*, *id*). The remaining steps in the above recursive formula remain the same. We could not establish analytical models to calculate *P*_*sc*_ for the remainder of the failure models using Markov relationships in time and space, and the respective analysis is based on simulations only.

### Hybrid chain

In a hybrid chain, a redundant peer is provided to every *m*^*th*^ node, as shown in Fig 3. The distance between the primary node and its redundant peer is denoted by *d*_*r*_. $d_{r}=1\;\text{m}$ is sufficient for independent and uncorrelated links to both nodes. To calculate the probability of successful packet delivery *P*_*hc*_ over *n* nodes of a hybrid chain, we augment the matrices *X*_*k*_, *A*, and *P*_*D*_ to include the respective entries for redundant peers along with the nodes of the primary chain, i.e.,

1. *X*_*k*_ is now of size *n*_*r*_ = *n* + ⌊(*n* − 1)/*m*⌋ to accommodate extra nodes. The notations are a bit complex in this scenario to accommodate extra peer for every *m*^th^ node. Although, we are still calculating the probability for each node to be a preferred forwarder, i.e., it has received the packet by any of the previous nodes but none of the other nodes closer to the destination has received the packet. The probability of being a preferred forwarder associated with the redundant nodes is inserted at every *im* + (*i* − 1), 1 ≤ *i* ≤ ⌊(*n* − 1)/*m*⌋ position of *X*_*k*_, i.e., *X*_*k*_(*im* + (*i* − 1)) contains the probability entry for redundant peer of node *im*. The remainder of the entries, i.e., *X*_*k*_(*i* + ⌊*i*/*m*⌋) is the probability entry for node *i*, 0 ≤ *i* ≤ *n* − 1, in the primary chain. *X*_0_ will be initialized in a similar manner to that described above for simple chain.
2. Similarly, matrix *A* is *n*_*r*_ × *n*_*r*_, and the probability of packet reception at node *i* when the transmitter is node *j* and nodes closer to the destination do not receive the packet is given as follows. P denotes the set of nodes in the primary chain, and R denotes the set of redundant nodes.
  $$\begin{array}{c} a_{IJ}=\{\begin{array}{cc} P_{i-j}\prod_{l=i+1}^{j+M}\left(1-P_{l-j}\right)\prod_{lm}\left(1-P_{lm-j}^{*}\right), & i,j\in P;\\ P_{i-j}^{*}\prod_{l=i}^{j+M}\left(1-P_{l-j}\right)\prod_{lm}\left(1-P_{lm-j}^{*}\right), & \begin{array}{c} j\in P,\\ i\in R; \end{array}\\ P_{i-j}^{*}\prod_{l=i+1}^{j+M}\left(1-P_{l-j}^{*}\right)\prod_{lm}\left(1-P_{lm-j}\right), & \begin{array}{c} j\in R,\\ i\in P; \end{array}\\ P_{i-j}\prod_{l=i}^{j+M}\left(1-P_{l-j}^{*}\right)\prod_{lm}\left(1-P_{lm-j}\right), & i,j\in R. \end{array}, \end{array}$$
  where $P_{i}^{*}=P(R,\sqrt{{\left(id\right)}^{2}+d_{r}^{2}})$, *M* = min{*T*(*r*), *n* − *j*}, *j* < *lm* ≤ *j* + *M*, *l* = 1, 2, …, and *I* and *J* are defined as follows,
  $$\begin{array}{c} J=\{\begin{array}{cc} j+\lfloor j/m\rfloor ,0\leq j\leq n-1, & j\in P;\\ jm+(j-1),1\leq j\leq \lfloor (n-1)/m\rfloor , & jm\in R. \end{array} \end{array}$$
  $$\begin{array}{c} I=\{\begin{array}{cc} i+\lfloor i/m\rfloor ,j\leq i\leq j+M, & i\in P;\\ im+(i-1),j<im\leq j+M, & im\in R. \end{array} \end{array}$$
  In our algorithm, a redundant peer will only be a preferred forwarder if the respective primary node will not receive the packet along with the nodes closer to the destination. Additionally, when a redundant node transmits, its peer in the primary chain is allowed to receive the packet, i.e., for i=j,j∈R and i∈P, $a_{IJ}=\hat{P}\prod_{l=i+1}^{j+M}\left(1-P_{l-j}^{*}\right)\prod_{lm}\left(1-P_{lm-j}\right)$, where *I* and *J* are given in the above expressions and $\hat{P}=P\left(R,d_{r}\right)$. If none of the nodes receive the packet in this case, the probability of being a preferred transmitter again in the next round will be $a_{IJ}=\left(1-\hat{P}\right)\prod_{l=i+1}^{j+M}\left(1-P_{l-j}^{*}\right)\prod_{lm}\left(1-P_{lm-j}\right)$. Here, *i* = *j* and i,j∈R.
3. *P*_*D*_ is also a vector of size *n*_*r*_, where *P*_*D*_(*i* + ⌊*i*/*m*⌋) = *P*_*n*−*i*_, 0 ≤ *i* ≤ *n* − 1 and $P_{D}\left(im+\left(i-1\right)\right)=P_{n-im}^{*},1\leq i\leq \left\lfloor \left(n-1\right)/m\right\rfloor$.

A list of variables and their respective definition is given in Table 1. The steps to update *X*_*k*_ are the same as given in Eq (10), and the probability of successful packet delivery over *n* nodes of a hybrid chain is similar to Eq (11), i.e.,

$$\begin{array}{c} P_{hc}=\sum_{k=1}^{n_{r}T_{R}}P_{D}X_{k}. \end{array}$$ (12)

Similar to the case of a simple chain network, if all nodes are assumed to have *iid* failures with probability *f*, we augment *P*_*i*_ in the formulation of matrices *A* and *P*_*D*_ given above as *P*_*i*_ = *P*(*R*, *id*)(1 − *f*), $P_{i}^{*}=P(R,\sqrt{{\left(id\right)}^{2}+d_{r}^{2}})\left(1-f\right)$, and $\hat{P}=P\left(R,d_{r}\right)\left(1-f\right)$. The remaining steps in the above recursive formula remain the same.

## Numerical analysis

Thus far, we have established the expressions for reliability, i.e., probability of successful packet delivery for different chain network topologies. The most important issue is now to validate the derived expressions before using them to understand the performance of multihop chain networks. To validate the expressions, we simulated the scenarios in MATLAB. A simple simulation generating random events according to the link and failure probabilities used in the reliability expressions is thus used to assess the accuracy of the theoretical models. Note that in this paper we focus only on the opportunistic routing, although MAC related delays would undoubtedly affect our results. While having an appropriate MAC-layer protocol to go with the opportunistic routing is crucial, it is equally important to check that if opportunistic routing can provide the potential and promise of acceptable performance in wake of excessive failures of rural environment, before such a MAC-protocol is developed.

### Validation of theoretical models

We simulated the packet reception at the nodes of simple and hybrid multihop chain networks according to the algorithm presented in Fig 2 using MATLAB. Random events were generated according to the packet delivery probabilities over wireless links given in Section Reliability of a Wireless Link and the node failure probabilities discussed in Section Mathematical Representation of Node Failures. For Markov failures, the previous state of the nodes, i.e., active or failed, was used to generate node failure events for the present step of the opportunistic routing. Only nodes in the active state can receive and forward. The node closest to the destination in the range *T*(*r*) from the transmitter that has received the packet was chosen as the next forwarder. Multiple non-interfering transmissions are possible, giving priority to the nodes closer to the destination. The transmission count was also saved and updated for each node, and when it reaches the re-transmission limit *T*_*R*_, the packet is dropped and the transmission count for the respective node is reset to zero. Over a thousand packets were transmitted in each trial over a distance of 100 km with different inter-nodal spacings. Each simulation result shown in the figures is averaged over 10 simulation trials.

To validate our calculations of probability of successful packet delivery for traditional best ETX path routing [20], i.e., *P*_*etx*_ given in Eq (14), we also simulated the algorithm in MATLAB. In this case, the best ETX path is found beforehand, and the random events of node failure and packet delivery over a link are generated only for the specific nodes in the path. Packets are dropped if an intermediate node reaches the re-transmission limit *T*_*R*_ without successfully delivering the packet to a forwarder. Ten trials, each with a thousand packets, were performed to estimate the probability of successful packet delivery and effective throughput of the best ETX path. All simulation results are shown with error bars representing a single standard deviation in each upper and lower direction.

For the analysis and simulation, the propagation model parameter values were chosen as follows: $f_{c}=2.4\;\text{GHz}$, ${\text{EIRP}}_{\left(\text{dBm}\right)}=36\;\text{dBm}$, $10\log_{10}{\left(G_{r}\right)}_{\left(\text{dB}\right)}=6$, $\sigma_{\epsilon }=8\;\text{dB}$, and h=9m, which is the average height of a utility pole. The parameters used in the link probability expression are $N_{0}=10^{-21}\;\text{W}/\text{Hz}$, BW=22MHz, and R=54/22bits/s/Hz, which are the specific values for 802.11g. Details about the choice of parameters are given in the related sections. The node failure model parameter values were arbitrarily chosen to be *f* = 10^−2^, *f*_*t*_ = 0.6, *f*_*s*_ = 0.5, and *f*_*ts*_ = 0.7. The re-transmission limit *T*_*R*_ = 3. A greater *T*_*R*_ will result in a higher probability of successful packet delivery across the network. The transmission range *T*(*r*) is defined in terms of outage probability, i.e., *P*(*R*, *id*)≥0.1, *j* ≤ *i* ≤ *j* + *T*(*r*), where *j* is the transmitter. All chain networks analyzed in this study spanned over 100 km with different inter-nodal spacings *d*.

**Fig 4:**
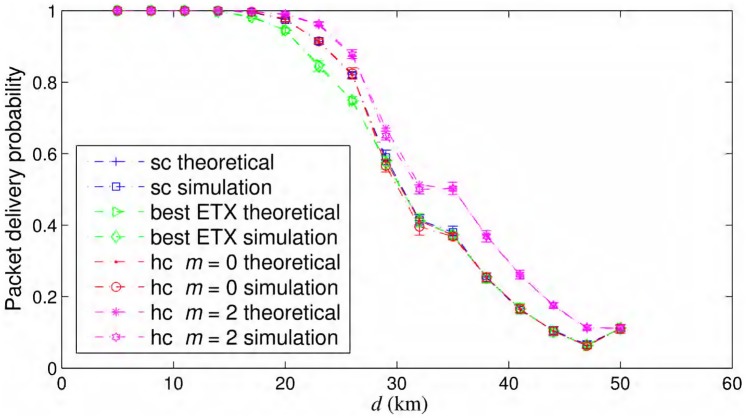
Validation of theoretical models. Analytical and simulated packet delivery probability over simple chain (sc) and hybrid chain (hc) networks with *iid* node failures with *f* = 0.01.

As shown in Fig 4, the simulations for the simple chain, hybrid chain with different values of *m*, and for the best ETX routing are in good agreement with the respective theoretical results. We considered *iid* node failures with *f* = 0.01 for all the cases shown in Fig 4. The best ETX path has the lowest probability of packet delivery over 100 km. Although, as the inter-nodal distance increases, there are less and less number of potential forwarders at each opportunistic routing step in simple and hybrid chain and with one potential forwarder, simple chain with opportunistic routing is the same as best ETX path as shown in Fig 4.

The probability of successful packet delivery is the best for the hybrid chain with *m* = 2 among the cases shown in Fig 4. At d=50km, the hybrid chain also becomes a simple chain with only one intermediate node besides source and destination and there is no redundant node. All probability curves converge for such setting as shown in Fig 4. Moreover, as noted previously, the hybrid chain with *m* = 0 becomes a simple chain, and the respective probabilities of packet delivery match well in Fig 4. The effects of selection of *m* are discussed later in Section Hybrid Chain Parameter.

The simulation results presented in Fig 4 are shown with error bars representing a single standard deviation in each upper and lower direction. Although not shown in this paper due to space constraints, the probability of successful packet delivery exhibits a similar trend, i.e., the best probability is found for a hybrid chain with *m* = 2, and the worst is found for the best ETX path among the topologies considered in Fig 4 when only outages are considered and without considering node failures.

### Node failures

**Fig 5:**
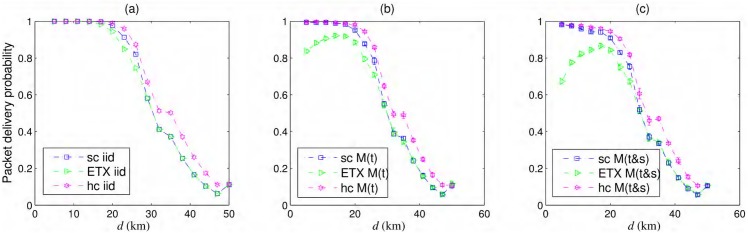
Packet delivery probability for different node failure models. (a) iid—*iid*, (b) M(t)—Markov in time, and (c) M(t&s)—Markov in time and space failures, for a simple chain (sc), best ETX path, and hybrid chain (hc) with *m* = 2.

We compared the effects of the different node failure models discussed in Section Mathematical Representation of Node Failures on the probability of successful packet delivery over different chain topologies and the best ETX path. As expected, the effects of node failures become worse when we consider correlation in time and space, as shown in Fig 5. The hybrid chain with *m* = 2 is the most robust against all types of failures among the options shown in Fig 5. It has a reasonable, although relatively smaller, probability of packet delivery for correlated failures in time and space, whereas the best ETX path behaves worse for correlated failure specially for smaller inter-nodal distances, i.e., when chain contains more nodes and consequently more failures. As also shown in Fig 4, as the inter-nodal distance increases, the opportunistic routing used in simple chain also has one potential forwarder at each step and its performance is the same as that of best ETX path. This fact is visible in all three plots of Fig 5. Moreover for *d* = 50, all three plots of Fig 5 show the convergence of all probability curves in similar manner as in Fig 4. In this case, the network has 3 nodes including source and destination and no redundant node and no forwarder diversity. For smaller inter-nodal distances, the simple chain also shows reasonable resilience against failures better than best ETX path.

Note that we have theoretical models for calculating the probability of successful packet delivery for *iid* node failures in Fig 5(a). The remainder of the failure models are simulated for all topologies. Error bars on the curves of the simulated results presented in Fig 5(b) and 5(c) show a single standard deviation in each upper and lower direction. With *f*_*t*_ = 0.6, the errors persist for *f*_*t*_/(1 − *f*_*t*_) = 1.5 forwarding steps on average, and with *f*_*ts*_ = 0.7 and *f*_*s*_ = 0.5, the errors on average affect approximately 1-2 successive nodes. The failures correlated in time and space are used to stress-test the hybrid chain, as the redundant nodes are also affected by the failures in neighboring nodes and might not be able to provide a bypass route. We expect that for failures affecting more nodes than average, the redundant nodes would not be a sufficient solution for providing reasonable reliability. Nevertheless, opportunistic routing and the use of redundant nodes provide considerably enhanced reliability for multihop chain networks.

### Hybrid chain parameter

**Fig 6:**
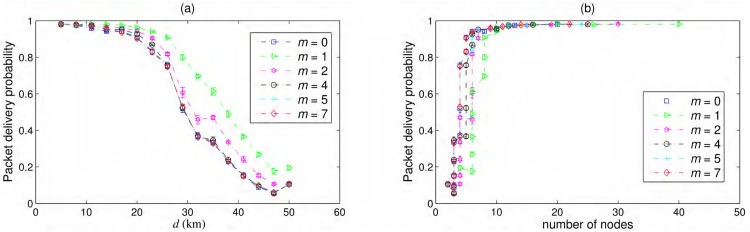
Packet delivery probability for different values of hybrid chain parameter *m*. (a) Packet delivery probability vs. inter-nodal distance and (b) packet delivery probability vs. number of nodes in the network. Failure model used here is Markov in time and space.

An interesting issue is to look for the effects of selection of hybrid chain parameter *m* over the performance of the network. As discussed before, a hybrid chain with *m* = 0 is a simple chain. When *m* = 1, the hybrid chain becomes a double chain with redundant nodes for every intermediate node between source and destination. The double chain will have the maximum probability of packet delivery or reliability among the considered topologies as shown in Fig 6(a). As expected, the probability of packet delivery for a hybrid chain with *m* > 2 falls between the probability curve of the hybrid chain with *m* = 2 and that of a simple chain in Fig 6(a). For *m* larger than the number of intermediate nodes in the network, the hybrid chain is the same as the simple chain as also shown in Fig 6(a) specially for *m* ≥ 5 for larger inter-nodal distance or smaller number of network nodes. At *d* = 50 km, all probability curves converge in Fig 6 except that of the double chain as it still has one redundant node in the network contributing in improved probability of packet delivery.

Although, double chain shows the best probability of packet delivery among all alternatives but it also requires most number of nodes which also means that it is the most costly topology. In Fig 6(b), we show the probability of packet delivery vs. number of nodes to understand the cost of improved performance. Interestingly the left-most knee of the curves, typically interpreted as the best trade off, consists of the points from *m* = 0 and *m* = 7 curves which basically means that no redundant nodes were used in the network as the total number of nodes are smaller than 7. Depending on the performance requirements and budget, Fig 6(b) can provide interesting guideline about network planning. Fig 6 is plotted for failures correlated in time and space but similar curves can be obtained for other failure models.

### Impact of routing overhead on throughput and delay

Multiple ACKs from all potential forwarders not only reduce the system’s capacity but also delay the delivery of the data packets. An important issue is to understand the unfavorable effects of opportunistic routing overhead over the performance measures of the network. Although, without modeling all protocol layers, it is not possible to calculate the effective throughput and packet delay of a network as specific protocol artifacts, such as channel access delays and queuing delays profoundly affect these measures. Moreover, the time slotted MAC, as required by our opportunistic routing algorithm, will also incur some overhead of synchronization, not modeled in the present study. Our analysis, however, allows us to get some clues about the adverse effects of opportunistic routing in a comparative manner. We calculate estimates of effective throughput and end-to-end delays for examples with opportunistic routing and compare them with similarly calculated estimates for cases with traditional routing. In practice, these values would most likely be worse than the ones reported in this paper for both routing regimes.

#### Effects on delay

For both types of chain networks, i.e., simple and hybrid, the average end-to-end delay Δ is defined as

$$\begin{array}{c} \Delta =\frac{\sum_{k}kP_{\delta }X_{k}}{\sum_{k}P_{D}X_{k}} \end{array}$$ (13)

where *P*_*D*_ and *X*_*k*_ are defined in Sections Simple Chain and Hybrid Chain for simple and hybrid chains. *P*_*δ*_ is a size *n* vector similar to *P*_*D*_, and each element is defined as *P*_*δ*_(*i*) = *δ*_*i*_ *P*_*n*−*i*_. *P*_*n*−*i*_ is the probability to reach destination from node *i* as given in Section Simple Chain. *δ*_*i*_ is the time taken in one step of opportunistic forwarding when node *i* forwarded the packet. For a packet size of 1 KB and standard 802.11 ACK size of 14 bytes, $\delta_{i}=8\left(1024+14\left(\min\left\{T\left(r\right),n-i\right\}+\left\lceil \min\left\{T\left(r\right),n-i\right\}/m\right\rceil \right)\right)/\hat{R}$, where $\hat{R}$ is the allowed data rate in bits/s. The quantity ⌈min{*T*(*r*), *n* − *i*}/*m*⌉ slightly over-estimates the number of redundant nodes within the range *min*{*T*(*r*), *n* − *i*}. A practical implementation would also require some guard times between the slots allocated to different transmitters, further increasing the step time.

We compared the mean delay of our opportunistic forwarding scheme with the traditional best ETX (expected transmission count) path routing algorithm [20]. *P*_*etx*_ is the probability of successful delivery of a packet over a multihop chain network via the best ETX path over *K* hops, and it is given as:

$$\begin{array}{c} P_{etx}=\max_{i}{\left[\sum_{j=0}^{T_{R}-1}P_{i}{\left(1-P_{i}\right)}^{j}\right]}^{K} \end{array}$$ (14)

where *P*_*i*_ = *P*(*R*, *id*) or when considering *iid* node failures, *P*_*i*_ = *P*(*R*, *id*)(1 − *f*). In this case, the step time is given as $\delta_{etx}=8\left(1024+14\right)/\hat{R}$ for a packet size of 1 KB. The average end-to-end delay for the best ETX path routing Δ_*etx*_ is given as,

$$\begin{array}{c} \Delta_{etx}=K[\sum_{j=0}^{T_{R}-1}P_{i}{\left(1-P_{i}\right)}^{j}\left(j+1\right)\delta_{etx}] \end{array}$$ (15)

where *K* is the number of hops to the destination and *P*_*i*_ is the probability of delivering a packet to the next hop.

**Fig 7:**
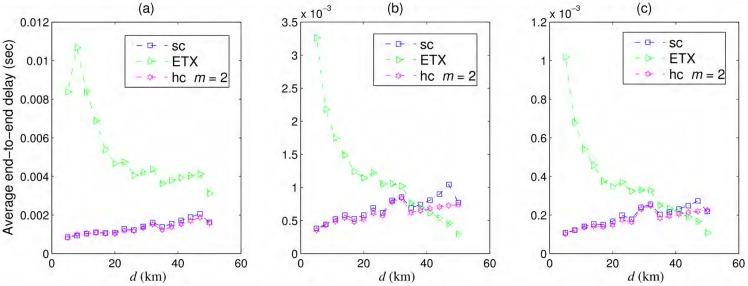
Average end-to-end delays. Average end-to-end delays experienced by delivered packets via the simple chain (sc), best ETX path, and hybrid chain (hc) with *iid* node failures using (a) 802.11b, (b) 802.11g, and (c) 802.11n.

The average end-to-end delays experienced by delivered packets over the simple chain, best ETX path, and hybrid chain with *m* = 2 are shown in Fig 7 for *iid* node failures using 802.11/b/g/n nodes. For 802.11b, R=11/22bits/s/Hz. For 802.11n, R=173/20bits/s/Hz. Link probabilities are calculated using Eq (9), using *N*_*T*_ = *N*_*R*_ = 4 and $f_{c}=2.4\;\text{GHz}$ for 802.11n. Note that delays are calculated from the theoretical models and there are no error bars shown in Fig 7. ITU-T G.114 recommends a maximum of a 150 ms one-way latency with less than 1% packet loss for VoIP. The values reported in our results are well within the ITU limits for some inter-nodal distances with a considerable margin for delays in practical scenarios which might be worse than the values shown here. The results are very encouraging and provide strong motivation for further exploration.

As number of hops to the destination increases, the best ETX path shows the general trend of reaching destination faster, although, the longer but weaker links may require more re-transmissions resulting in longer delays for some settings as also shown in Fig 7. Moreover, the end-to-end delay values for opportunistic routing are better than that of best ETX path in most of the scenarios which indicates that the additional delays due to multiple ACKs are not adversely affecting the overall performance. Multiple ACKs in opportunistic routing reduces the need of packet retransmissions which incurs even longer delays as shown in our results. Similarly, for longer inter-nodal distances, the need of re-transmission grows in simple and hybrid chain as well yielding longer average delays when compared with the topologies with smaller inter-nodal distances. Moreover, additional delays of opportunistic routing cause longer average delays than best ETX path for larger inter-nodal distances, or networks with lesser but weaker number of links. We also note that the average latency of the delivered packets is directly affected by *T*_*R*_, the allowed transmission count, and does not depend on the rate of node failures, which actually affects the packet delivery rate. We do not expect that the latency values will change considerably for other failure models.

#### Effects on throughput

In this analysis, we also simulated the wireless multihop chain operation in MATLAB, primarily to validate the theoretical models. The MATLAB simulation experiments generated random events according to the packet delivery and node failure probabilities given above. The simulations are also very useful in providing estimates for data rate or throughput. The details of setting up the simulations are discussed in Section Validation of Theoretical Models. We transmitted over a thousand packets in each simulation experiment to estimate the effective throughput *ρ*, as given below.

$$\begin{array}{c} \rho =\frac{\text{Packets}\;\text{received}\times \text{Packet}\;\text{size}\;\text{(bits)}}{\text{Time}\;\text{taken}} \end{array}$$ (16)

To compare the performance of the proposed opportunistic forwarding scheme with that of the traditional best ETX path routing approach, we also estimated the effective data rate of the latter through simulations under the same operational settings as used for the former. Note that the simulation and theoretical models in this analysis do not consider specific protocol artifacts, such as channel access delays, queuing delays, and so on. The throughput and mean end-to-end delay estimates calculated in this analysis can be considered to be the best expected results.

**Fig 8:**
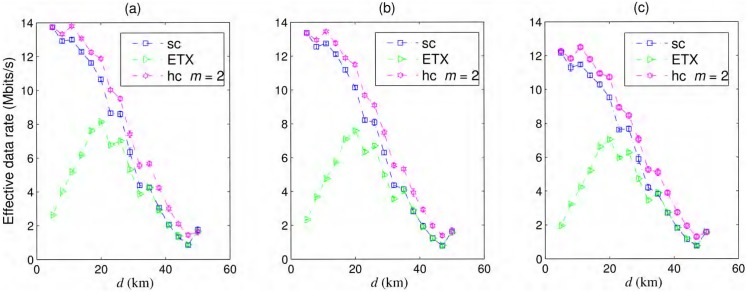
Effective data rates. Effective data rates for a simple chain (sc), best ETX path, hybrid chain (hc) with (a) iid—*iid*, (b) Markov(t)—Markov in time, and (c) Markov(t&s)—Markov in time and space failures.

The effective data rates for a simple chain, best ETX path, and hybrid chain with *m* = 2 are shown in Fig 8 for all types of node failures considered in this paper. Interestingly, the hybrid chain provides the best effective throughput among all the cases discussed here. We expected that the capacity of the multihop chain would be reduced with the addition of redundant peers, as there would be more interfering nodes, but as the hybrid chain is more resilient to failures, it also has the best throughput. Some jumps in the curves appear when *T*(*r*) changes from a higher value due to a smaller inter-nodal spacing to a smaller value due to a larger inter-nodal spacing. With smaller *T*(*r*), the waiting time for all ACKs to be received will become smaller, and more data can pass through within this period. When there is not a large difference in the probability of packet delivery for both cases, the one with smaller *T*(*r*) have the higher throughput.

For the case of the best ETX path, we considered the interference range where *P*(*R*, *d*) ≥ 0.1. The effective throughput of the best ETX path is quite poor when the network consists of many hops or for smaller inter-nodal distances even for *iid* node failures due to the longer time taken in traversing the many hop path. For longer and weaker links, higher packet drop is the major reason behind low effective throughput for all cases. Moreover, the best ETX path also show smaller throughput for correlated node failures, as shown in Fig 8, and the hybrid chain showed reasonable performance even for failures correlated in time and space. The best throughput gained by the hybrid chain, with *iid* failures, over 100 km is approximately 14 Mbits/s, whereas $\hat{R}/3=18\;\text{Mbits}/\text{s}$ for 802.11g.

In the practical scenarios, the throughput would be adversely affected by the protocol artifacts, dynamic wireless media, etc. Although, we do not expect that the comparative merit of opportunistic routing over traditional routing will go away as successful delivery of packets strongly depends on the resilience of routing protocol in wake of intermittent failures.

### Cost

The most important question to answer now is the cost per delivered bit. The cost of deploying and maintaining the multihop chain network is directly proportional to the number of nodes. Additionally, note that the poles used in our analysis are very small compared to the towers required for long-distance WiFi links. Moreover, if the nodes are deployed along roads and on already erected utility poles, then the cost can even be lower, along with a reduced maintenance cost, because of the accessibility of the network site; however, in this case, we would need to consider issues such as interference from power lines. We also do not need high gain directional antennas for multihop backhauls, thus reducing the cost even further.

**Fig 9:**
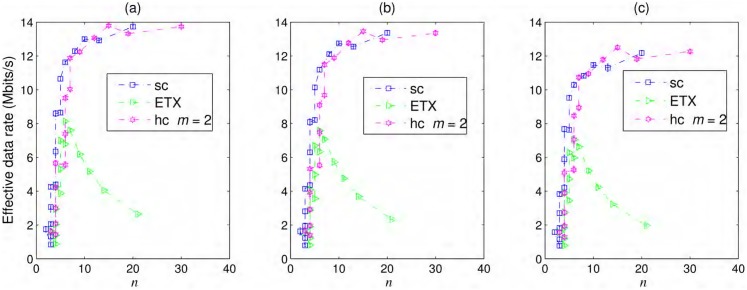
Effective throughput versus number of nodes. Effective throughput for a simple chain (sc), best ETX path, and hybrid chain (hc) with *m* = 2 for (a) *iid* node failures, (b) failures correlated in time, and (c) failures correlated in time and space.

Fig 8 is redrawn in Fig 9 with the x-axis now showing the number of nodes *n* for simple and hybrid chains and for the best ETX path. For approximately 5 802.11g nodes to span 100 km, we can obtain a net throughput of 12 Mbits/s for *iid* node failures. Although, it is not fair to compare our theoretical results with a practical deployment, the throughput requirement in the Ashwini network is 384 Kbits/s, and the capital cost of each node is $5000. The cost per Kbit is thus $26. If we assume that the WiFi equipment cost is $100 for each node including the router [4], antenna, and solar panel etc., and that the nodes are mounted on existing poles, the cost per Kbit in our deployment is $0.05. Moreover, it is not necessary for all nodes of the multihop chain to be operating all the time, and the inherent redundancy of the wireless broadcast medium can manage failures via an opportunistic routing approach. However, for long-distance point-to-point links, a node failure means no connectivity.

We also conducted the experiment shown in Fig 9 using an 802.11b configuration, i.e., R=11/22bits/s/Hz, although the resulting figure is not shown here. The effective data rates follow similar trends to those in Fig 9, but they are considerably lower for 802.11b nodes. The maximum effective throughput is over 4 Mbits/s for 802.11b.

**Fig 10:**
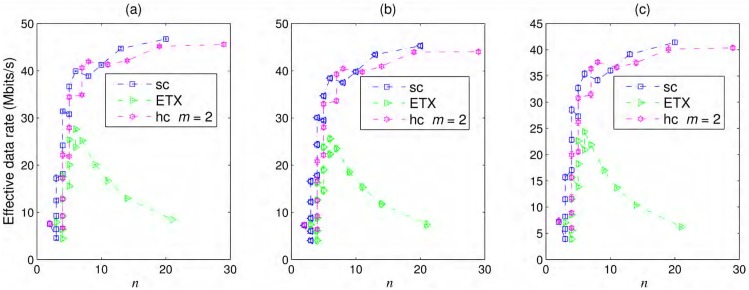
Effective throughput versus number of 802.11n nodes. Effective throughput for a simple chain (sc), best ETX path, and hybrid chain (hc) with *m* = 2 using 802.11n nodes with (a) iid—*iid*, (b) Markov(t)—Markov in time, and (c) Markov(t&s)—Markov in time and space failures.

The probability of packet delivery over a link using the MIMO antenna configuration given in Eq (9) was used to calculate the effective throughput versus number of nodes for 802.11n shown in Fig 10. We assumed that *N*_*R*_ = *N*_*T*_ = 4, R=173/20bits/s/Hz, BW=20MHz, and $f_{c}=2.4\;\text{GHz}$. 802.11n (or more advanced 802.11ac) nodes are widely and economically available throughout the world, and they could be the best choice for WiFi rural backhauls due to the higher effective throughput. The knees of the cost curves shown in Figs 9 and 10 can be regarded as the best operating points. Adding more nodes after the knee shows a relatively small improvement in effective throughput. According to Figs 10 and 7, over 40 Mbits/s throughput can be achieved by approximately 5 802.11n nodes when the average end-to-end latency for delivered packets remains under the VoIP limit for connectivity over 100 km.

### Cost vs. Pole length

Thus far, all of the above analyses considered 9 m poles and ${\text{EIRP}}_{\left(\text{dBm}\right)}=36\;\text{dBm}$, which is the maximum allowed transmission power. A list of estimated costs for poles in USD (2007) are given in [4]. The cost of a large tower is typically a couple of orders of magnitude higher than that of a normal utility pole. Moreover, alternate power supplies, such as solar panels, would be more appropriate in a rural operational setting, and the choice of allowed maximum transmission power would also influence the overall cost. A smaller power supply with fewer solar cells, in general, costs less than a relatively larger solar power source. Moreover, FCC allows to use up to ${\text{EIRP}}_{\left(\text{dBm}\right)}=52\;\text{dBm}$ in 2.4 GHz band for fixed point-to-point links by using higher gain antennas and reducing 1 dBm power for every 3 dB gain. Higher gain antennas are generally more expensive but there are other factors too, such as, type of antenna, which have more significant influence over its price. The selection of transmit power and antenna with appropriate gain is an important design parameter effecting cost and performance. The following question thus arises: what would be the best selection for pole height and allowed transmission power to minimize cost for a given operational environment?

The objective function to minimize is *Cost/Throughput*. For a homogeneous network, such as the one considered in our analysis, cost is the number of nodes times the cost of a single node, which in turn depends on the cost related to the pole height, the power supply, antenna, and other miscellaneous components, e.g., CPU, which are considered constant for this analysis. Throughput is measured through the simulation experiments described above in saturation conditions. Note that effective throughput can only be calculated considering the whole protocol stack. Here, we only have upper bounds for throughput to work with.

The parameters of the simulations for 802.11n remain the same as that used in Figs 10 and 7 except EIRP and antenna height, i.e., *N*_*R*_ = *N*_*T*_ = 4, R=173/20bits/s/Hz, BW=20MHz, and $f_{c}=2.4\;\text{GHz}$. The hybrid chain parameter *m* = 2, and the allowed number of re-transmissions *T*_*R*_ = 3. All other simulation parameters are same as that of Section Numerical Analysis. Antenna pole height is selected as 2, 9, 15, 24, 30, and 45 m. The cost for pole heights is taken from Table 1 of [4]. We arbitrarily added the cost for 2 m pole as $35/- and assumed that the cost of a 9 m pole was same as that of a 10 m pole in [4]. EIRP is set as 33, 36, 42, 52 dBm with antenna gains of 6, 6, 15, 30 dB respectively. Considering the low cost of modern solar panels, we considered a constant cost model for power supply, with $4.87 as the cost of 1 W supply. A constant cost of $200/- is considered for the router and miscellaneous parts. For antennas with gains 15 dB and 30 dB, extra costs of $10/- and $20/- are added respectively. It should be noted that the cost estimates used in this analysis are not absolute values. It really depends on when and where the components are purchased. Our Google search for the price of antennas, solar panels, and masts shows variations in different countries. For the masts, we decided to remain conservative and use prices reported in [4] in 2007. They may be different or more probably higher now. For a real deployment, the analysis should be done with current and local estimates including shipping costs for items purchased from other countries or cities.

For each pole height and power option, we varied the number of nodes in the chain (simple and hybrid) around the knee of the effective throughput vs. number of nodes curves as shown in Figs 9 and 10 to obtain the minimum number of nodes/throughput value among simple and hybrid chains. This value was then multiplied by the total node cost for a particular combination of pole height and power supply options.

**Fig 11:**
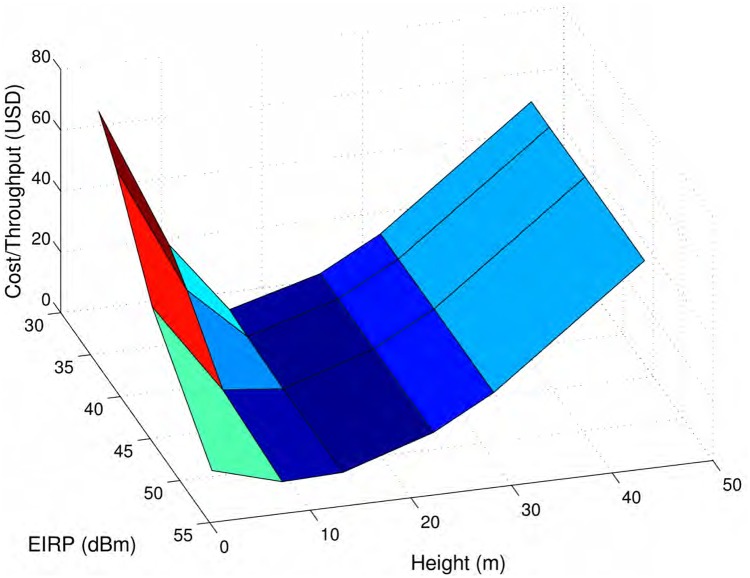
Cost/Throughput. Cost/Throughput for multiple pole heights and allowed EIRP for *iid* node failures Pole height of 9 m with EIRP of 52 dBm yields the minimum Cost/Throughput.

Fig 11 shows the Cost/Throughput with *iid* node failures. An interesting insight from Fig 11 is that higher transmission power improves the cost/throughput function for very small poles, i.e., the increase in cost of using higher gain antennas is over compensated by improvement in data rates. For longer poles, using higher powers yield similar results as the lower transmission power because the throughput is already better. The effect of pole height over the objective function is much more pronounced than the effect of power supply. For higher pole heights, the cost of poles inflates the overall objective function although only 2 nodes were required for connection. For low pole heights, such as 2 m, the number of nodes become excessive to tip the balance. With pole height of 2 m and EIRP of 33 dBm, over 40 nodes were required to give the best performance of the operating scenario. According to Fig 11, pole height of 9 m with EIRP of 52 dBm gives the optimum Cost/Throughput. As we have discussed before, if existing utility poles are used, this option would become even more desirable due to lower cost.

An important issue to note here is that our theoretical model is unable to capture the distortions, i.e., terrain and earth curvature affects, obstacles, etc., for longer links. In this analysis, longer links appear to be considerably better than their expected real-life performance. It would really be interesting to recalibrate the signal models specially for very long links using real testbed values and redraw Fig 11 to find the optimum operational settings. Very similar results were obtained when the cost/throughput function was plotted for 802.11g nodes and for other failure models; although the figures are not shown here.

### Reality check using field data

The radio propagation models are generally limited in their ability to accurately represent the signal propagation in wireless media even in open spaces of rural areas [21, 22]. Consequently, the accuracy of the performance results based on these models are also limited. On the other hand, field experiments can give us only a snapshot of the performance space for a particular experimental setting and for the specific channel state at the experiment instant. It is usually not possible to reproduce exactly same conditions and consequently same results in field experiments. The theoretical models, however, allow us to comprehensively analyze the operating conditions and it is impossible to study optimal settings or relative merit of specific scenarios without the help of theoretical models. The important question is: how to make conclusions, from the theoretical study, meaningful and relevant for reality?

A comparative study of the performance predicted from theoretical models and the performance witnessed in the field experiments can given us an idea about how to interpret the theoretical results. The discrepancy between predicted and real performance for some operational settings can let us study the effects of the prediction error and devise generic solutions applicable to other operational settings as well, which is the main goal of this section.

**Fig 12:**
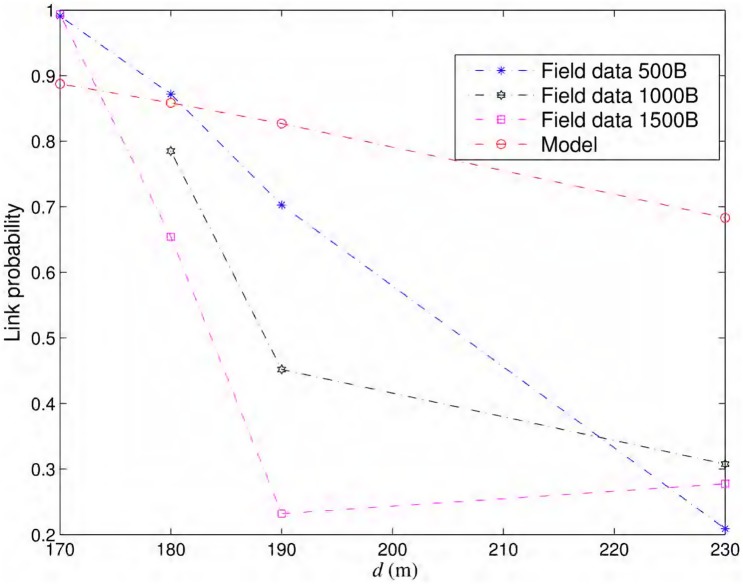
Link probabilities vs. distance. Link probabilities from the field experiments and also from Eq (7) for the same conditions.

In this section, we use packet loss data [23] for 802.11b routers for different transmitter-receiver distances collected in a wide uncultivated field with an unobstructed line of sight, far from buildings, cell phone antennas and power lines. Fragmentation, RTS/CTS, retransmissions (ARQ) and dynamic rate switching were disabled for the experiments and different fixed data rates of 1, 2, 5.5 and 11 Mbits/s, with three fixed frame lengths (500, 1000, 1500 bytes), were used in these experiments. 200,000 packets were transmitted in each experiment. Fig 12 shows probabilities of successful packet transmission for links of different lengths for 11 Mbits/s and all packet sizes calculated from the statistics of 200,000 transmissions. We also show the link probabilities predicted by the Eq (7) under similar conditions. Specifically, the selected parameters are: $f_{c}=2.4\;\text{GHz}$ and BW=22MHz for 802.11b. ${\text{EIRP}}_{\left(\text{dBm}\right)}+10\log_{10}{\left(G_{r}\right)}_{\left(\text{dB}\right)}=61.5$ and h=1m as described for the experiment setup [24]. Shadowing variance is $\sigma_{\epsilon }=8\;\text{dB}$ for the open rural areas. Noise spectral density is selected as $N_{0}=10^{-21}\;\text{W}/\text{Hz}$ to yield typical noise floor value of -100 dBm for 802.11 receivers [17]. Note, that the link probability calculation of Eq (7) does not incorporate the packet size so the Fig 12 shows one model based link probability curve along with three curves calculated from the field data for each packet size. Smaller packets mostly, but not always, yield better link probability in field experiments, although, the model of Eq (7) does not capture this effect.

As shown in Fig 12, the theoretical model generally overestimates the link probabilities for longer distances. The model may provide a good fit for relatively smaller distances as shown in Fig 12, but it does not capture accurately the sharp descent of link strength in practice. This fact is also reported in literature [21] and we have observed in all data sets of field experiments. Figures are only shown for 11 Mbits/s data sets. Interestingly, the really long distance WiFi links, over 10’s of kms long, have been observed to behave closer to theory in terms of error rates versus Signal-to-Noise Ratio (SNR) curves [5].

**Fig 13:**
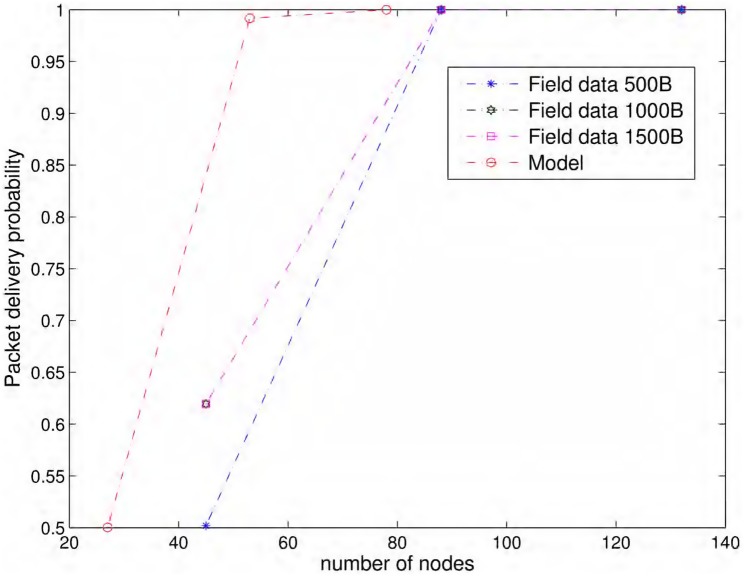
Packet delivery probabilities vs. number of nodes. Packet delivery probabilities over 10 Km vs. number of nodes from the field experiments and also from the theoretical model under same conditions with *iid* node failures.

As a consequence of overestimation of link probabilities shown in Fig 12, the path probabilities also seem to be better in general when calculated using the propagation model. For the same operating conditions, Fig 13 shows the packet delivery probabilities calculated over a path length of 10 Km for different number of equidistant nodes. In field experiments, the lowest packet delivery probability recorded for 11 Mbits/s and all packet sizes is at a transmitter-receiver distance of 230 m. We calculated the path probabilities of packet delivery for three inter-nodal distances of 230 m, 115 m, and 76.6 m. Since we do not have link probabilities for all inter-nodal distances from the field experiments, we used linear interpolation/extrapolation to calculate the needed values. As shown in Fig 13, the first case with lowest number of nodes, i.e., the largest inter-nodal distance of 230 m, have the lowest path probability. This case has only one forwarder in each opportunistic routing steps as the probability of reaching the next node 460 m away is almost zero. The next case with half of inter-nodal distance, i.e., 115 m has very strong likelihood of packet delivery over 10 km with multiple potential opportunistic forwarders. At 230 m, the link probabilities on 1000 bytes and 1500 bytes curves are very close to each other yielding the same performance for lowest number of nodes in Fig 13 and both path probability curves appear merged. The link probability on 500 bytes curve is the smallest at 230 m yielding the respective lowest path probability in Fig 13.

When Eq (7) is used for similar conditions, link distance of 390 m yields packet delivery probability of 0.2088. In this case, we selected three cases of inter-nodal distances of 390 m, 195 m, and 130 m. As shown in Fig 13, model-based probability of packet delivery over a 10 km path is much better than the one calculated from the field data. The model predicted path probability of 0.9918 with 53 nodes, whereas the field data yield path probability of 0.6627 with 45 nodes for 1000 and 1500 bytes’ packets and even lower for 500 bytes’ packets as it has the lowest link probability at 230 m as shown in Fig 12. The discrepancy between model-based and field data-based predictions is because of overestimation of link probabilities by the propagation model. In Fig 13, approximately 50% additional nodes, than those predicted by the model, are required for 10 km path to yield path probability close to unity using field data.

As a general rule, we can expect that a practical deployment should include additional nodes than predicted by the model to compensate for the overestimation of probabilities. To explore this issue further, we assumed that *S*(*d*)_*real*_ = *S*(*d*)_*model*_ − *ω*, where *S*(*d*)_*real*_ is the actual received signal power at distance *d* and *S*(*d*)_*model*_ is the signal strength predicted by the propagation model given in Eq (1). *ω* is the overestimation of received signal strength by the model. All three quantities are assumed to be in dBm. Ideally *ω* should be modeled as a random noise taking both positive and negative values but since overestimation of signal strength is very costly for our work and could lead to an impractical and unconnected network, we assume that *ω* ≥ 0. Substituting $S{\left(d\right)}_{\left(\text{dBm}\right)}$ in Eq (6) with *S*(*d*)_*real*_, we get the following expression:

$$\begin{array}{ccc} P(R,d) & = & 1-\mathrm{Pr}(\epsilon_{\left(\text{dB}\right)}<10\log_{10}(10^{3}\left(2^{R}-1\right)\sigma^{2})+\omega -\alpha +\beta \log_{10}\left(d\right))\\ & = & 1-Q(\left\{10\log_{10}(10^{3}\left(2^{R}-1\right)\sigma^{2})+\omega -\alpha +\beta \log_{10}\left(d\right)\right\}/\sigma_{\epsilon }) \end{array}$$ (17)

**Fig 14:**
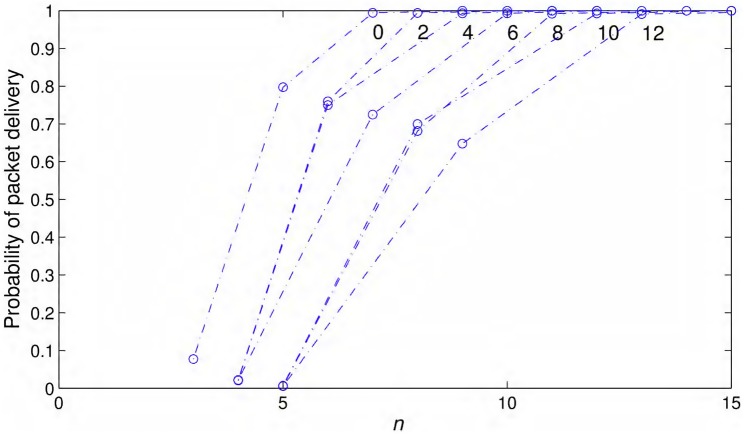
Propagation model error. Probability of packet delivery versus number of nodes for a simple chain with *iid* failures with propagation model error in dBm.

All the other variables are the same as defined for Eq (7). Fig 14, shows the probability of packet delivery with *iid* node failure using a simple chain over a 100 km path for 802.11g settings, i.e., $f_{c}=2.4\;\text{GHz}$, R=54/22bits/s/Hz, and BW=22MHz. ${\text{EIRP}}_{\left(\text{dBm}\right)}=36\;\text{dBm}$, $10\log_{10}{\left(G_{r}\right)}_{\left(\text{dB}\right)}=6$ and h=9m. Shadowing variance is $\sigma_{\epsilon }=8\;\text{dB}$, noise spectral density is selected as $N_{0}=10^{-21}\;\text{W}/\text{Hz}$ and node failure probability is *f* = 10^−2^. As expected, as the error *ω* is increased from 0 dBm to 12 dBm, the number of nodes needed to yield path probability close to unity increases. No error or ω=0dBm case needs 7 nodes whereas ω=12dBm case almost doubles the number of nodes to 13. The results presented in Fig 14 depend on the operating conditions but, as a rule of thumb, we can expect to use nearly double the nodes predicted by the model to provide a safe margin for possible overestimation. With this doubling we can raise the cost of the network to $0.1 per Kbit as opposed to $0.05 per Kbit depicted in Section Cost which is still well under the cost of long distance WiFi links, i.e., $26 per Kbit (cf. Section Cost). Pilot link measurements should also be conducted before the actual deployment to determine the extent of discrepancy between the model predictions and real values.

## Related work

This section presents a brief survey of the state of the art in the areas of rural communications and opportunistic routing.

### Rural communications

There has been many initiatives around the world to resolve the issue of rural connectivity. Some important efforts directly relevant to our paper are discussed in Section Introduction, such as, aerial networks of Google and Facebook and projects using long distance WiFi links [3–6], e.g., the DGP (Digital Gangetic Plains) network and FRACTEL deployed in rural India [5, 25]. In the DGP network [4, 5], a centrally located node serves as the gateway connectivity for 15 nodes sparsely deployed to connect villages up to a distance of 80km. The network also optimizes power using a Wake-on WLAN that remotely power nodes on demand. The end devices are personal computers (PCs) employed for IP telephone services such as VoIP. FRACTEL deployed sixteen nodes to provide broadband access and support multimedia applications [25] using both short and long distance links. It utilizes a central node to provide gateway connectivity from a single point to connect multiple buildings across many villages. Its’ unique feature arbitrates reliable QoS support for VoIP and video-based services via TDMA-based MAC layer re-design. An example deployment of Fractel is the Ashwini Project in Andhra Pradesh [4].

Although, it is not fair to compare our theoretical results with a practical deployment, the throughput requirement in the Ashwini network is 384 Kbits/s, and the capital cost of each node is $5000. The cost per Kbit is thus $26. In Section Cost, our results show that with approximately 5 802.11g nodes to span 100 km, a net throughput of 12 Mbits/s can be obtained for *iid* node failures. If we assume that the WiFi equipment cost is $100 for each node including the router [4], antenna, and solar panel etc., and that the nodes are mounted on existing poles, the cost per Kbit in our deployment is $0.05. The cost may double, i.e., $0.1 considering the error between received power and its prediction through models as shown in the previous section. Our results show strong potential of a cost-effective alternative in the form of mutihop WiFi chains to the long distance WiFi links and an economical overall solution for rural connectivity.

Moreover, it is not necessary for all nodes of the proposed approach of multihop chain to be operating all the time, and the inherent redundancy of the wireless broadcast medium can manage failures via an opportunistic routing approach. However, for long-distance point-to-point links, such as in Ashwini network, a node failure means no connectivity.

Intel has also developed a Rural Connectivity Platform (Intel^®^ RCP) to provide robust, long distance (up to 100Km) WiFi based backhaul connections based on the research done in [3]. In rural Africa, less than 1% of people have access to the Internet [26]. The Macha network is one of the largest rural mesh installations deployed in southern Zambia [19] and consists of 52 nodes. In total, the network has approximately 100 active devices connected to the nodes or access points, with 100-150 daily users. The central part of the entire Macha network is the campus of the John’s Hopkins Malaria Institute at Macha (MIAM), with 11 mesh nodes. One of them works as a gateway and is connected via two Very Small Aperture Terminal (VSAT) satellite receivers with Committed Information Rates (CIR) between 32 kbits/s and 128 kbits/s to the outside world.

Similar technologies have been used in several large projects related to communication in rural areas such as VillageTelco project [27], Wray Project in England [28] (moved to fibre in 2010), AirJaldi Network in India [7], Pebbles Valley Mesh Network in South Africa [29], Sengerema Mesh Network [30] and Tegola Mesh [31]. Similarly, an 802.11a/g commercial network is deployed in rural New Zealand by University of Waikato and a local ISP [22]. Experimental networks using WiFi have also been deployed across rural UK for research regarding the use of UHF white spaces [32]. Finally, a small multi-radio mesh network called WiBACK [26] has been established in Lesotho. It offers voice and WiFi/Internet access in parts of Lesotho. A single gateway supplies Internet access for the entire mesh which is setup as an energy-aware network. Each node needs no more than l0W to operate. Independence from power grids is an important aspect in a rural area, as power outages may occur more often. Therefore, it is preferable to use renewable energy sources, such as, wind or solar energy generators, to provide power to the nodes.

The concept of Delay Tolerant Networking (DTN) is used in [33, 34] where mechanical backhauls and disconnected networks are used to provide a cost-effective solution to connecting remote sites over tens of miles but prohibit the use of real-time applications, such as, VoIP, interactive video, etc.. These types of networks are also called opportunistic networks. Instead of connecting the remote villages and sites to the data network via expensive links or cables, DakNet [33] talks about installing wireless nodes on buses and vehicles which can retrieve and transfer data to and from a village access point while passing through it.

Moreover, there has been number of efforts in utilizing GSM/cellular base stations for rural connectivity. These base station are within $10,000/- [35] and mechanisms are proposed to make them extremely energy efficient for low power consumption [36] and be able to use GSM white spaces without effecting primary users [35] in order to utilize unused licensed spectrum. Huawei also launched energy efficient EasyGSM base station for rural areas in 2009 which could be used with only solar power.

### Opportunistic routing

There is a large body of work in opportunistic routing. Most of these approaches can be divided into five different categories: Geographic, link state aware, probabilistic, optimization-based and cross layer opportunistic routing. Methods that are based on the nodes’ locations, such as CBF [37] and GeRaF [38], are in the first category. This category is also relevant to the rural connectivity application discussed in this paper. There are around 15 different algorithms described in [12] under this category and they differ in terms of implementation details. Any of the algorithm suitable to the operating conditions can be used and as long as the routing exploits the inherent redundancies of wireless medium, we can expect to achieve improved reliability in comparison to the conventional routing approaches.

The second category, which is also relevant to the rural operational scenarios, includes proposals that aim to improve the network performance by taking into account the link quality, bandwidth and/or remaining energy in the opportunistic routing design, such as ExOR [10], MORE [39], etc. Prior work such as Epidemic and Spray and Wait [12], are in the third category. They tried to tackle the problems of nodes’ mobility and changing network topology using statistical network characterization and online link availability and quality prediction. The fourth category covers the proposals that use optimization tools such as convex programming, game theory and machine learning theory to formulate the problem of opportunistic routing. Examples are like Consort [40], AdaptOR [41] and SMAF [42]. The methods in the last category are about cross layer opportunistic routing, such as CORMAN [43] and MTOP [44], where information are exchanged between the Network layer, the Physical (PHY) layer and/or the MAC layer in order to get more accurate routing metrics measurements and scheduling-aware routing decisions that could be implemented in real network devices. [12] provides an extensive review of these protocols.

## Conclusion

In this paper, the reliability of wireless chain networks is studied in terms of the probability of successful packet delivery over multiple hops spanning 100 km. The effects of the wireless propagation medium are considered through empirical models, and node failures are modeled as independent events and as correlated events in time and space. Our analysis shows that it is possible to deploy an economical wireless chain network spanning a distance of hundreds of kilometers with reasonable resilience against uncertain radio propagation and node failures. Improved robustness of chain networks can be achieved by using opportunistic forwarding methods and by adding redundancies in network topology. Networks with redundant nodes also exhibited robustness against various failure situations.

In this initial study, we show mathematically that wireless multihop chain networks could be a promising solution for providing connectivity to remote regions as an alternative to long-distance WiFi. We also compared link probabilities calculated from the field experiments to that of the model under similar conditions and their respective probabilities of packet delivery over a 10 km path. The model overestimated the probabilities and consequently additional nodes are needed to yield satisfactory performance than predicted by the model. Considerable effort is still required to determine the feasibility of multihop backhaul connections using more realistic models and finally with real hardware. The present paper is thus only served as a preliminary study for a more comprehensive research project of deploying a pilot broadband network in rural Pakistan.

Moreover, a study of various rural communication projects around the world shows that a more practical approach would probably include merging of various technologies, such as, cellular (GSM, 3G, etc.), WiFi, WiMax, dynamic aerial networks, etc. Availability of specific equipment at economical prices depends on the location. After 3G roll-out, the leftover GSM equipment was available at much cheaper prices. Similar trends will be observed in future. For a real deployment in future, we will be looking at the merging of different technologies suitable to the terrain and the budget. Also, the issues regarding plug-and-play capability will be explored to cater for dynamic expansion and adaptability of the network.
